# Electro-Magnetism in Intermittent Fever

**Published:** 1846-10

**Authors:** 


### ELECTRO-MAGNETISM IN INTERMITTENT FEVER.

An esteemed correspondent, to whom we have heretofore been indebted for interesting facts, communicates the following cases of intermittent fever cured by electro-magnetism:

I had been laboring under intermittent fever from August, 1845, until April, 1846. Several of the best physicians had been consulted without benefit. Quinine, the usual remedy, seemed to have lost its effect; the chill frequently returning in three or four days after its suppression. In April, being at Louisville on business, the weather suddenly turned cold with rain, and immediately I began to experience the peculiar nervous depression, which every one similarly situated feels in such weather, and, calling on a medical friend, we amused ourselves some time in his office with an electro-magnetic battery. Immediately I found myself free from all nervous debility, and felt nothing of the kind for some hours. On my return, in reflecting on the circumstance, I determined on the next occasion of the paroxysm to test the efficacy of a battery at home. In a few days the opportunity was afforded, and the chill was completely arrested in six minutes; and even after the nails were blue and the hands cold above the wrists. I have had no chill since, and have taken no quinine.

Some time after this, Mr. B., who had been laboring under intermittent fever for the last three months, called on me. The usual remedies had been prescribed without effect; the chills continued to return at short intervals, and when I saw him he had had a return each day for more than a week. I administered a cathartic of blue mass and compound extract of colocynth, but from some cause without effect; and expecting a chill next morning, I placed him under the influence of the battery, and the time passed by without any chill, though some evidences of its approach were apparent.

In my own case, the biliary organs were in good condition, and the paroxysm seemed to recur more in virtue of habit than otherwise; but in the case of Mr. B. the liver and spleen were both evidently and badly deranged.

Do not these cases authorize us to expect something from electricity in protracted intermittents? In recent cases the remedy would not seem to me to promise so much. I hope physicians will give it a trial in some of their obstinate cases, and communicate their experience to the public.

Shelbyville, Ky., September, 1846.

